# *Scotty*: lattice coincidences in the Protein Data Bank

**DOI:** 10.1107/S2059798326005723

**Published:** 2026-06-24

**Authors:** Airlie J. McCoy, Lawrence C. Andrews, Herbert J. Bernstein, Randy J. Read

**Affiliations:** a Cambridge Institute for Medical Research, Department of Haematology University of Cambridge The Keith Peters Building, Hills Road CambridgeCB2 0XY United Kingdom; b Ronin Institute for Independent Scholarship 2.0 9515 NE 137th Street Kirkland WA98034 USA; c Fresh Pond Research Institute c/o Brookhaven National Laboratory NSLS II Upton NY11973 USA; Lund University, Sweden

**Keywords:** non-isomorphism, PDB annotation, oligomerization, paracrystalline arrays

## Abstract

The application *Scotty*, implemented within the *Phasertng* codebase, was used to perform a PDB-wide analysis of lattice coincidences. Using a broad definition of lattice coincidence, the number of distinct lattice clusters is approximately half the total number of crystallographic PDB entries. In over one thousand lattice clusters entries are reported in different space groups, consistent with pseudo-symmetric variation within a common lattice framework. Space-group frequencies computed at the lattice-coincidence level update those obtained by entry-based counting, and more accurately reflect priors for novel crystal forms. Combining lattice clustering with sequence identity and deposited oligomeric annotations reveals multiple cases of inconsistent biological assembly assignments among structures sharing near-identical lattices, suggesting an opportunity to improve annotations. The survey also identifies a range of protein systems forming extended *in cellulo* paracrystalline arrays, including storage, sequestration, toxin and membrane-associated proteins, an understudied area of structural biology. Overall, the results demonstrate that lattice-level analysis provides a valuable perspective on macromolecular self-association.

## Abstract 2

*Scotty* supports crystallographic comparisons of lattice coincidences, the interpretation of oligomeric assemblies and paracrystallinity.

## 1. Introduction

Searching the Protein Data Bank (PDB) for crystals with similar macromolecules arranged within a similar lattice is not trivial. Even in the simplest case of the same macromolecule in the same crystal form, crystallographers in different laboratories may choose different settings. Non-isomorphism will make the search more difficult, as unit-cell differences between related lattices can be very high; for example, the 10 Å change in a unit-cell dimension in HIV-1 reverse transcriptase (Esnouf *et al.*, 1998 ▸). The problem compounds if there has been a breakdown in one or more symmetry relationships within the lattice, so that the space groups also differ; even the crystal system can vary.

To improve searches for similar lattices we have developed *Scotty*, a software tool that searches the PDB to find lattice matches regardless of space-group setting, annotation or subgroup relationship, and allowing significant perturbations in lattice distances and angles, and other types of non-isomorphism such as skewed translations and small-molecular rotations (McCoy *et al.*, 2026 ▸). The search uses NCDist, a Niggli reduced cell distance metric (McGill *et al.*, 2014 ▸), to find nearest-neighbour lattices in the Niggli reduced cell space, followed by structure-factor intensity correlation (CC_*I*_) to filter for underlying atomic distribution matches. By identifying ‘coincident lattices’, where crystal forms share the same fundamental three-dimensional macromolecular packing, we can recognize recurring higher order assemblies. These can be grouped to form ‘lattice clusters’ where members of a cluster share three-dimensional intermolecular associations that build the crystal.

Lattice clusters can be evidence of consistent oligomerization of homologues. Crystal lattices habitually incorporate the protein solution oligomerization state (Gaber & Pavšič, 2021 ▸). Protein oligomers usually possess point-group symmetry compatible with a space-group symmetry (such as Schönflies *C*_2_, *D*_2_), and the oligomer effectively reduces the number of unique crystal contacts required for lattice formation (Wukovitz & Yeates, 1995 ▸). This mechanism increases crystallization propensity, and engineering oligomerization has even been proposed as a technique to encourage crystallization (Banatao *et al.*, 2006 ▸). That oligomeric symmetry is incorporated into crystallographic lattices can be seen indirectly in the altered space-group propensities for proteins that oligomerize versus those that are monomers in solution (Gaur, 2021 ▸).

In some specialized cases the entire crystalline lattice has biological relevance, such as in the case of insulin (Asai *et al.*, 2022 ▸). While rare, functionally crystalline proteins exist across all kingdoms of life, linked to storage, secretion, structural rigidity or protection (Doye & Poon, 2006 ▸; Schönherr *et al.*, 2018 ▸). *In vivo* crystalline arrays are generally understudied and many remain uncharacterized.

Biologically relevant interfaces tend to recur across different crystal forms, bury larger surface areas, display shape and electrostatic complementarity, and exhibit evolutionary conservation of key residues. Quantitative criteria estimating the likelihood that a given interface corresponds to a stable oligomer in solution are implemented in software tools such as *PISA* (Krissinel & Henrick, 2007 ▸) and *EPPIC* (Duarte *et al.*, 2012 ▸). *ProtCID* (Xu & Dunbrack, 2020 ▸, 2011 ▸; Xu *et al.*, 2008 ▸) develops the idea of interface recurrence as evidence of biological relevance by clustering protein interfaces across PDB crystal structures at the Pfam domain level, to identify recurring protein–protein, domain–peptide, nucleic acid and ligand interactions and thereby generate hypotheses about biologically relevant assemblies.

Better identification of matching and near-matching lattices could strengthen approaches such as *ProtCID* by separating true recurrence of an interface from simple repetition of the lattice. If the same macromolecule crystallizes repeatedly in crystal forms falling in the same lattice cluster, this in itself is little evidence for biological relevance. In contrast, detection of related lattices among homologous proteins is highly informative. Finding lattice clusters could therefore act both as a filter, down-weighting redundant observations from essentially identical crystal forms, and as a discovery tool, highlighting conserved domain contacts in homologues where sequence, domain architecture and crystal packing have varied but the same interface persists. This would be particularly useful for weak oligomeric interfaces, which are difficult to distinguish from crystallization-induced contacts and which are often missed by biological assembly annotations or interface-prediction tools (Xu & Dunbrack, 2020 ▸).

In this study, we use *Scotty* to survey the PDB to group the entries into lattice clusters and probe the resulting classification for physical and biological properties, giving examples of how the method can deepen analysis of difficult-to-study interactions.

## 2. Survey of the PDB for coincident lattices

A survey of lattice coincidences within the PDB archive was undertaken with the *Scotty* software (McCoy *et al.*, 2021 ▸, 2026 ▸). *Scotty* was used to probe the PDB metric-space index built from each crystallographic structure in the 20260101 PDB snapshot with each structure in the same snapshot (all-on-all).

### 2.1. Measuring lattice coincidences

The *Scotty* software uses the NCDist (Niggli-cell distance) metric to find matches between Niggli reduced unit cells, a canonical representation of the lattice (unit cell) agnostic to space group and space-group setting. Matches are then filtered by CC_*I*_, which quantifies how similar the underlying atomic arrangements are via the structure factors, either observed or calculated. Results are reported in terms of √CC (= 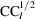), which is an estimate of σ_A_, the complex correlation between true and model structure factors, and is also the expected real-space map correlation. *Scotty* allows a speed optimization to avoid costly structure-factor calculations for CC_*I*_ calculation by allowing NCDist matches to be (conservatively) filtered by applying a (low) sequence-identity threshold between the match and the query.

The NCDist threshold for lattice matches was 5 Å. Lattice coincidences were filtered from the NCDist lattice matches returned from the PDB index for having at least one of the two criteria: either the √CC_*I*_ was over 0.4 or the sequence identity between the model and target was over 30%, a low threshold for indicating the same fold.

### 2.2. Correlation between NCDist and √CC_*I*_

**Figure 1:**
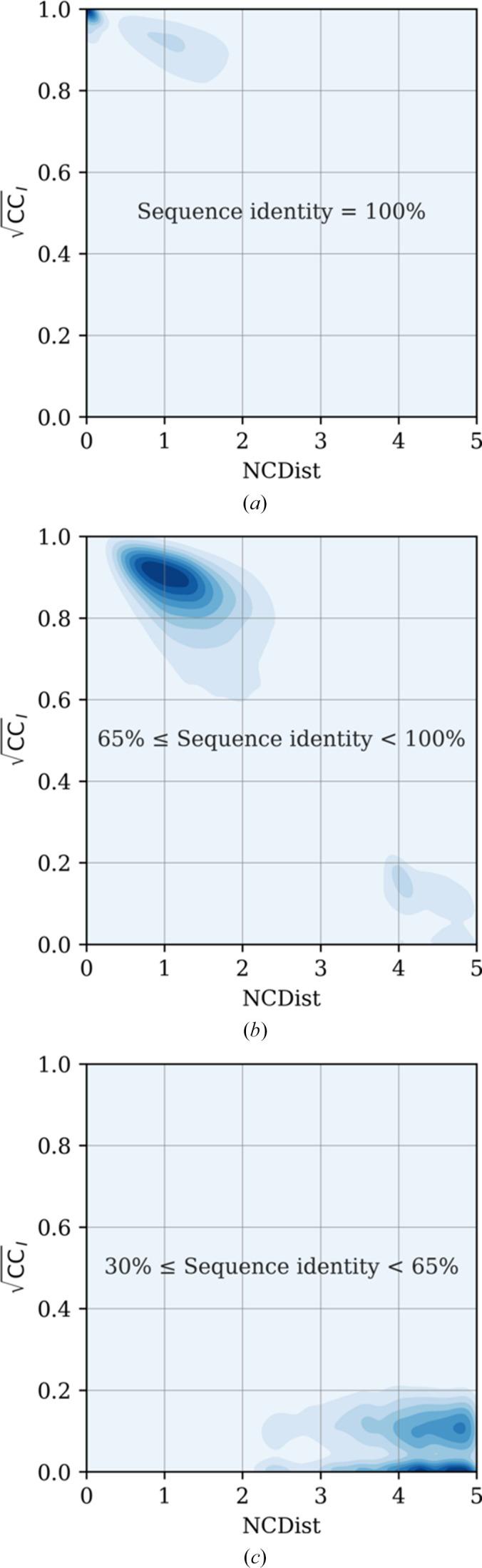
Kernel density estimates of the relationship between NCDist and √CC_*I*_ for lattice coincidences in PDB snapshot 20260101 by sequence identity. (*a*) Identical sequences (100% sequence identity), (*b*) moderate pairwise sequence identity (between 100% and 65%) and (*c*) low pairwise sequence identity (between 65% and 30%). In each panel, the distribution is shown as filled density contours, with darker shading indicating higher probability density.

To evaluate the link between lattice similarity and electron-density agreement, the joint distribution of NCDist and √CC_*I*_ was analysed across three sequence-identity regimes (Fig. 1 ▸).

(i) 100% sequence identity: shows a sharp peak at √CC_*I*_ = 1 and NCDist = 0 indicating indistinguishable electron-density maps.
(ii) 65% to 99% sequence identity: shows a broad anti­correlation as map agreement gradually degrades with growing lattice dissimilarity.
(ii) 30% to 65% sequence identity: shows a bimodal distribution spanning NCDist 3–5 Å with a primary peak at √CC_*I*_ ≃ 0 and a secondary peak at √CC_*I*_ ≃ 0.1, demonstrating that at least for some of these cases a lattice coincidence is weakly detectable. An important subset of this group (5.4%) has √CC_*I*_ > 0.4.

Cases in which there is a lattice match by NCDist < 5 Å and yet √CC_*I*_ ≃ 0 can arise for three reasons.

(i) Alignment artefacts: pairwise alignments can inflate identity scores near the 30% threshold, particularly in low-complexity regions or sequences of mismatched lengths.
(ii) Filter limitations: the sequence identity is assessed using only the longest polymer chain. Small proteins or large peptides can occupy lattice voids or associate differently and alter √CC_*I*_ without changing the underlying Niggli cell parameters. Note that the choice to only use the primary sequence is deliberate as, conversely, it is robust to false negatives due to low sequence identity of minor components.
(iii) Conformational differences: significant structural or conformational variations, even between identical macromolecules, can degrade √CC_*I*_ even when crystal-packing contacts remain unchanged.

### 2.3. Space groups with lattice coincidences

**Figure 2:**
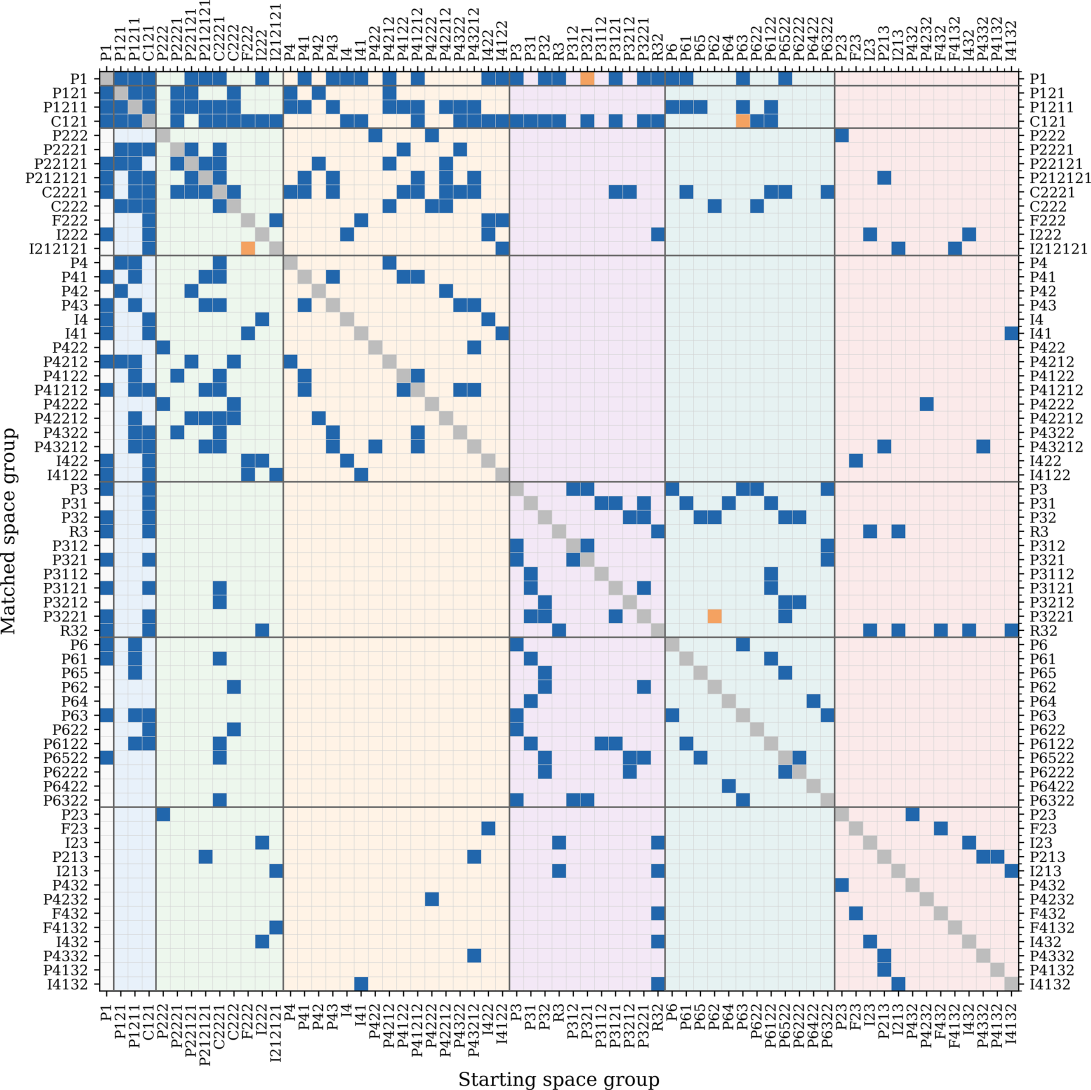
Matrix of space groups of lattice coincidences in PDB snapshot 20260101: probe space group (horizontal axis) and matched space group (vertical axis). Crystal systems for probe space group are coloured for clarity and boundaries between crystal systems are shown with a grey line for both probe and matched space groups. Grid positions are blue where the relationship is found in the data and orange where the relationship is missing but the reciprocal relationship is present; these isolated cases are due to numerical instability in the calculation of √CC_*I*_ at the selection boundary. Within the 87 404 mismatch pairs, 3174 entries matched with higher symmetry space groups in 30 821 pairs, 13 536 entries matched with lower symmetry space groups in 54 568 pairs, and 1068 entries matched with different space groups with the same number of symmetry operations in 2015 pairs.

Matches with √CC_*I*_ > 0.4 and differing space groups were identified. The matrix of space-group pairs (Fig. 2 ▸) is generally sparse. The matrix is nearly symmetric above and below the diagonal, which confirms that the procedure is reciprocal and stable: if *A* matches *B*, *B* matches *A*. Rare nonsymmetric entries are due to minor numerical border-crossing effects around the 0.4 inclusion threshold.

The matrix has a block-like structure, revealing that most space-group transformations stay within the same crystal system by gaining or losing a single symmetry operator within tetragonal, hexagonal or cubic systems, or from orthorhombic to monoclinic β = 90°.

Certain high-connectivity space groups act as operational ‘hubs’, linking families of subgroup/supergroup transitions. Low-symmetry space groups *P*1 and *C*2 are prominent in this role, often involving the loss/gain of more than one symmetry operator.

There are isolated matrix entries that are not explained by loss of symmetry but rather change of space-group screw-axis symmetry, for example *P*2_1_2_1_2_1_ to *P*2_1_2_1_2. These cases have more than one molecule in the asymmetric unit. Where the screw has been lost, it is substituted for translational non­crystallographic symmetry (tNCS) that can be characterized as a pseudo-centring operator; for example, a tNCS vector at (0, 0, ½). The ambiguity lies in the alternative selections of the space-group symmetry/noncrystallographic symmetry, which may not always have been made optimally in the deposited structure.

Our observations do not necessarily imply that there is an error in any of the analysed crystal entries. When clusters contain structures spanning different space groups, these variations need not be byproducts of poor data analysis or flawed processing. These crystallographic differences may be fully justified by the underlying data-merging statistics, indicating that the lattice-matching algorithm is successfully clustering genuine, physically real perturbations in the crystal lattice symmetry. Furthermore, even in specific instances where the data might be under-merged, or there are differences in space-group symmetry/noncrystallographic symmetry pairings, there is no reason *per se* to expect the resulting structural models to be incorrect in any biologically significant way.

### 2.4. Lattice coverage of PDB

**Figure 3:**
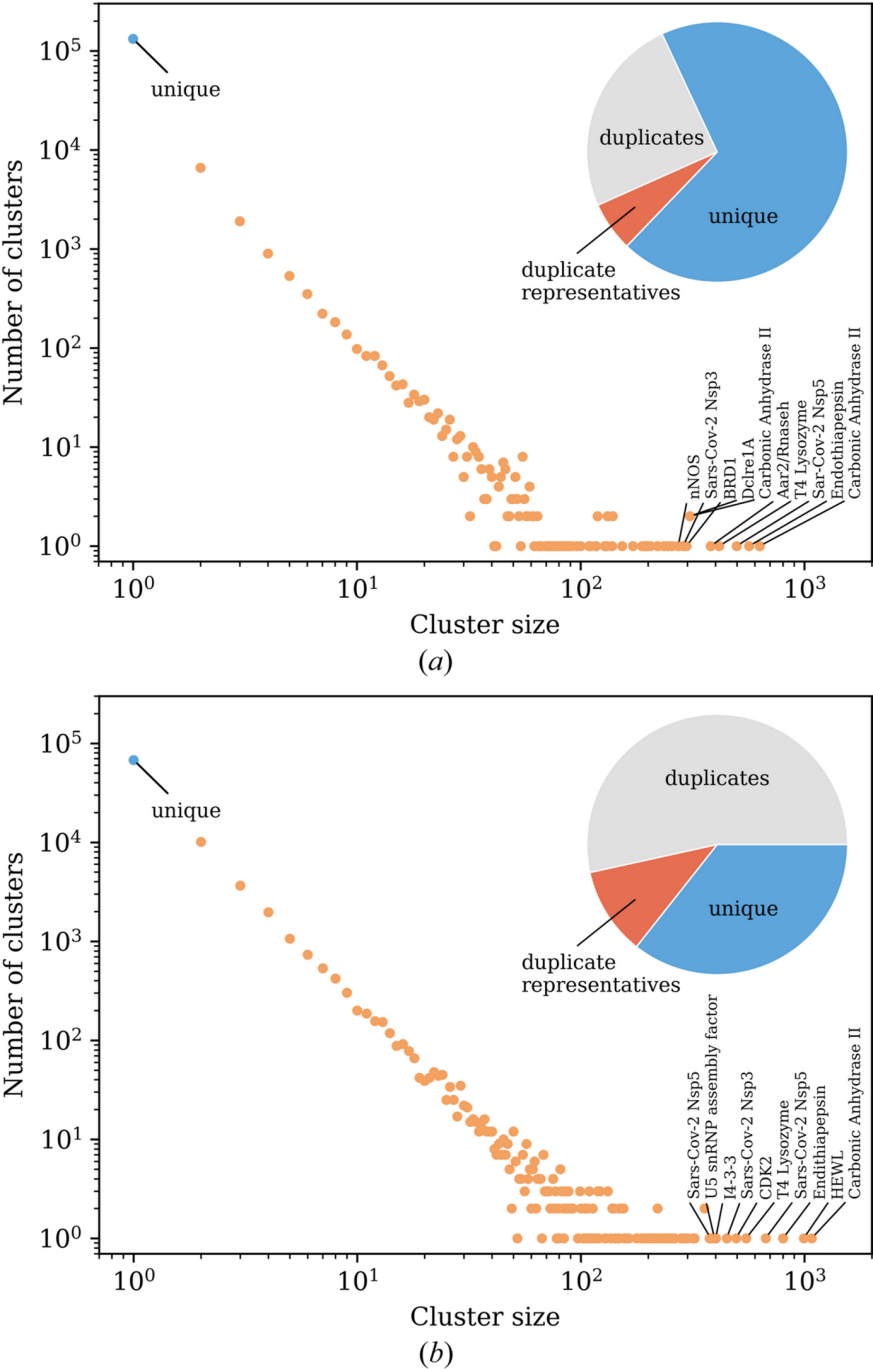
Effect of duplicate identification on the effective search space for 190 770 entries in PDB snapshot 20260101. The main panel shows that the number of clusters decreases approximately linearly with increasing cluster size on log–log scaling consistent with a power-law-like decay. A long tail corresponds to a small number of highly over-represented entries. The largest ten clusters are labelled. Inset: proportion of entries retained after collapsing each duplicated cluster to a single representative. (*a*) Duplicates defined as the same unit cell, same space group, same sequence and low r.m.s.d. without fitting, as described in the text. (*b*) Duplicates defined by lattice clustering, as described in the text.

PDB entries were clustered into lattice-coincident groups using an undirected graph where the edges represent the pairwise matches and then finding all connected components. The database reduced to 88 846 lattice clusters (Fig. 3 ▸), demonstrating that lattice diversity can be represented by approximately half of the entries in the PDB.

We compared the number and size of the lattice clusters generated by *Scotty* with the number and size of trivially isomorphous replicate groups in the PDB. To qualify as trivially isomorphous – a duplicate – entries had to contain the same macromolecules with 100% primary-sequence identity, share identical deposited space groups and settings, exhibit tightly matched deposited unit-cell parameters (within 1.5 Å on unit-cell edges and 1.5° on unit-cell angles and an overall NCDist < 1.0 Å) and display a full-atom r.m.s.d. of less than 2 Å calculated strictly without rigid-body fitting (McCoy *et al.*, 2026 ▸). Because this overlay condition relies entirely on unmodified deposited coordinates, the resulting count is sensitive to the historical archival issues, where identical structures were sometimes processed in inappropriately low-symmetry space groups or alternative coordinate frames (Wlodawer *et al.*, 2018 ▸, 2025 ▸; Dauter & Wlodawer, 2018 ▸). Fortunately, there has been recent work to remediate the PDB archive so that structural duplicates can be cleanly recognized in automated workflows. Constructing an undirected graph where edges represent verified duplicates, finding all connected components yielded a total count of 161 121 replicate clusters, meaning that lattice clusters offer considerably larger groups and a reduced lattice diversity compared with that offered by lattice duplicates (Fig. 3 ▸).

### 2.5. Lattice coincidences with multiple space groups

Out of the 88 846 lattice clusters, 1538 had two or more space groups (Table 1 ▸). Clusters containing four or more space groups were limited to short DNA or α-helical peptide segments. These clusters were caused by the propensity of DNA and α-helices to form end-to-end continuous helical stacking in crystals. The resulting diffraction is dominated by the shared helical molecular transform, inflating the √CC_*I*_. Excluding these pathological cases, multi-space-group variations occurred in 1442 clusters and were capped at a maximum of four space groups.

The 1442 clusters containing multiple space groups generated a total of 87 404 directed pairwise mismatches between entries. This tally is heavily skewed by a few exceptionally large clusters due to cross-combination scaling with 2 × *N*_1_ × *N*_2_, where *N*_1_ and *N*_2_ are the numbers in each space-group category: a large cluster containing one entry with an outlier space group (*N*_1_ = 1, *N*_2_ ≫ *N*_1_) contributes disproportionately to the total. The median cluster containing multiple space groups had only two space groups and three entries (*N*_1_ = 1, *N*_2_ = 2), contributing four pairwise mismatches to the total.

We further assessed the multiple space-group clusters with other validation tools.

#### 2.5.1. Under-merging (higher symmetry pairs)

When analysed via *POINTLESS* (Evans, 2011 ▸), 51% of the 3008 entries (out of 3174) with deposited experimental data were flagged as potentially under-merged. An additional 27% had metrics indicating under-merging that fell just short of internal software thresholds for being reported.

The remaining 22% of the entries with deposited data were not identified as having higher lattice symmetry, showing that the *Scotty* procedure is more sensitive to detecting coincident lattices than the lattice matching and merging methods implemented in *POINTLESS*.

That the PDB contains under-merged data has been known since the audit by Dauter *et al.* (2014 ▸), who characterized these crystallographic discrepancies as avoidable errors. The authors argued that while these lower symmetry models are technically valid, they obscure the true symmetry of the assembly, complicate crystal-packing analysis and introduce artificial structural clutter that hinders automated data harvesting.

#### 2.5.2. Over-merging/twinning (lower symmetry pairs)

*PDB-REDO* identifies twinning using *SFCHECK* (Vaguine *et al.*, 1999 ▸) twinning statistics followed by *REFMACR*-value statistics (Joosten *et al.*, 2014 ▸). Tested against *PDB-REDO* gold-standard flags, only 1.7% of the 13 536 entries matched with lower symmetry counterparts were annotated as twinned. Because this is far below the baseline PDB-wide average of 4.2%, classic crystal twinning does not explain these over-merged classifications.

### 2.6. Space-group statistics

Historical surveys of PDB space-group propensities consistently confirm a tripartite dominance of *P*2_1_2_1_2_1_, *P*2_1_ and *C*2. However, the specific reported percentages vary significantly across the literature (published between 1990 and 2021) depending on database size, inclusion criteria, curation and/or categorization (Table 2 ▸).

Space-group statistics for the PDB were calculated with one space group per lattice-coincidence cluster. Where a cluster had multiple space groups, the space group with the highest symmetry was taken to represent the cluster (Supplementary Table S1). This study finds that the space-group frequencies by lattice cluster closely match the frequencies of Chruszcz *et al.* (2008 ▸), but push *P*2_1_ even further towards equal frequency to *P*2_1_2_1_2_1_.

## 3. Lattice coincidences and quaternary structure

To evaluate the quaternary-structure information that lattice coincidences provide, we focused on crystals of single macromolecular protein entities (which excluded hetero-oligomers) from the 20260101 snapshot of the PDB archive. A structured query submitted to the RCSB PDB search API retrieved entries meeting the following criteria: (i) having all unit-cell dimensions greater than 5 Å, (ii) including one protein entity, (iii) excluding any nucleic acid polymer entities (DNA, RNA or hybrid), (iv) consisting of over 50 amino acids per assembly, (v) having only one deposited entity instance count and (vi) a polymer entity mutation count equal to zero.

The search returned 47 588 PDB identifiers in 6272 clusters. The assembly records of all entries in each of the 6272 clusters were extracted from the archive and were used to infer the oligomeric state of each entry.

### 3.1. Space-group statistics

Because biological oligomers generally have point-group symmetry, space groups *P*1, *P*2_1_, *P*2_1_2_1_2_1_, *P*4_1_, *P*4_2_, *P*4_3_, *P*3_1_, *P*3_2_, *P*6_1_, *P*6_5_, *P*6_2_, *P*6_4_ and *P*6_3_, which lack point-group symmetry operations (Schönflies *C*_2_, *C*_3_, *C*_4_, *C*_6_, *D*_2_, *D*_3_, *D*_4_, *D*_6_, *T* or *O*), are underrepresented (Table 3 ▸). However, a space group’s maximal point-group symmetry does not necessarily match the oligomeric symmetry, since crystal-packing interactions can impose additional symmetry elements: this is as true for oligomeric assemblies as it is for monomeric proteins.

### 3.2. Oligomers

The survey enabled us to checkpoint the consistency of annotation of oligomeric symmetry within the PDB archive.

To check PDB oligomeric annotation consistency, clusters were filtered using *MUSCLE* alignments to eliminate low-diversity groupings, where the internal sequence identity exceeded 95%. This left 304 clusters that had retained lattice coincidences through evolution. The 304 cases were equally divided between enzymes (158) and non-enzymes (147). Individual cases of lattice coincidences were examined on a case-by-case basis for biological relevance. We give four examples below.

#### 3.2.1. Keto–acid reductoisomerases

Keto–acid reductoisomerases from *Campylobacter jejuni* (PDB entry 7lat) and *Methanothermococcus thermolitho­trophicus* (PDB entry 7q03) with 50% sequence identity share an *I*23 lattice coincidence after applying a re-indexing operation (−*h*, *l*, *k*), with √CC_*I*_ = 0.42 and NCDist = 2.14 Å. The latter is annotated as a dodecamer (with point-group symmetry *T*) while the former is annotated as a dimer (with point group *C*_2_).

In *EPPIC* (Bliven *et al.*, 2018 ▸), both are identified as dimers. *PISA* (Krissinel & Henrick, 2007 ▸) identifies the latter as a dodecamer and the former as a dimer, which matches the annotation (and is likely the source of the annotation). However, a closer look at the interfaces interpreted by *PISA* for the two cases show that the differences are very marginal (Table 4 ▸).

In addition, the keto–acid reductoisomerase from *Bacillus anthracis*, with 57% sequence identity to that from *C. jejuni* and 54% sequence identity to that from *M. thermolitho­throphicus*, crystallizes in space group *I*222 with three monomers in the asymmetric unit. This crystal form has NCDist = 3.5 Å and √CC_*I*_ = 0.41 to that from *M. thermolithotrophicus*. It too is annotated as a dimer but shares the same dodecameric assembly within the lattice.

The coincident lattices suggest that the dodecamer is likely to be present in solution prior to crystallization.

#### 3.2.2. Cardiotoxin

Cardiotoxins are small, disulfide-rich, non-enzymatic proteins that disrupt cell membranes. Cardiotoxin γ (PDB entry 1tgx) and cardiotoxin IV (PDB entry 1ug4) from two different species of cobra, with 85% sequence identity, share a lattice coincidence after applying a re-indexing operation from *C*2 to *H*32 (−*h* + *k*, *h* + *k*, ⅓*h* − ⅓*k* − ⅓*l*) with NCDist = 2.228 Å and √CC_*I*_ = 0.49. *POINTLESS* does not identify the coincidence.

Cardiotoxin γ, the *C*2 crystal form, contains three molecules in the asymmetric unit arranged around a noncrystallographic threefold axis. This trimer has been proposed to represent a biologically relevant assembly, an ion-conducting trimeric pore, which is then stacked back-to-back by a crystallographic twofold axis to form a continuous hexameric channel. Cardiotoxin γ is annotated as a hexamer, and its pore-forming ability would be consistent with the known membrane-disruptive activity of cardiotoxins (Bilwes *et al.*, 1994 ▸). The structure of cardiotoxin IV has the same hexameric arrangement, but around the crystallographic threefold and twofold axes in *H*32, with one monomer in the asymmetric unit. Nonetheless, cardiotoxin IV is annotated as a monomer in the archive.

#### 3.2.3. Methionine adenosyltransferase

Across the four human methionine adenosyltransferase (MAT) structures with PDB entries 8oog, 2p02, 8xam and 2obv, a consistent picture emerges once the crystals are compared at the lattice level rather than by deposited space-group annotations alone. The three PDB entries 8oog, 2p02 and 8xam are structures of MAT2A, while PDB entry 2obv corresponds to MAT1A, a closely related paralogue expressed in adult liver with 86% identity to MAT1A. The MAT2A structure 8xam in space group *I*121 with two molecules in the asymmetric unit shares a lattice coincidence with the other three, all of which are in space group *I*222 with one molecule in the asymmetric unit, and therefore all four have the same crystalline assembly. However, PDB entry 8oog is annotated as a monomer, PDB entries 2p02 and 8xam as *C*_2_ dimers and PDB entry 2obv as a *D*_2_ tetramer. It is most parsimonious to infer that MAT1A and MAT2A share a common tetrameric structure in solution, with the tetramer consistently represented in the crystal lattice even when not explicitly annotated as such in the PDB.

#### 3.2.4. Ras-family GTPase

An intriguing case is provided by Ras-family GTPases. A lattice cluster of 86 structures (for example, PDB entries 7ycc, 5us4, 1lf0, 4l9w, 2cl0 and 3i3s), which includes H-Ras and K-Ras, and has pairwise sequence identities between 90% and 100%, have closely related hexagonal settings *H*32 (one molecule in the asymmetric unit), *H*3 (two molecules in the asymmetric unit) or *C*121 (PDB entry 7ycc, three molecules in the asymmetric unit). They are annotated variously as a monomer, dimer, trimer and hexamer. Despite this heterogeneity in space group and biological assembly annotation, the underlying crystallographic arrangement is a consistent recurrent hexamer. Ras has been shown to form nanoclusters on the plasma membrane, with each cluster containing on average ∼6–7.7 Ras molecules and having a radius of approximately 60 Å (Plowman *et al.*, 2005 ▸). The repeated observation of a conserved lattice across multiple Ras crystal structures with divergent sequences suggests that there is an energetically favourable set of intermolecular contacts compatible with nanocluster formation.

### 3.3. *In cellulo* paracrystalline arrays

Protein crystals can occur naturally *in cellulo* (McPherson, 1999 ▸), an observation dating back to the mid-19th century. The assemblies often lack the long-range periodicity required for classification as true crystals and are often termed paracrystalline arrays. Naturally occurring crystals and paracrystalline arrays are known to serve a large variety of specific biological roles (Doye & Poon, 2006 ▸). In archaea and bacteria, 2D crystals or paracrystalline arrays are well characterized, serving protective and structural roles. Helical assemblies of proteins are also known to be functionally important, enabling dense storage, dynamic organization and mechanical force generation across a variety of biological systems.

Paracrystalline arrays naturally lend themselves to study by X-ray crystallography; however, cryo-electron microscopy has also been utilized, for example in the study of ribosome microcrystals found in chick embryos (Nurullina *et al.*, 2024 ▸; Barbieri, 1979 ▸)

Interestingly, given the rarity of *in cellulo* 3D, 2D and 1D helical arrays, the clusters of lattice coincidences with divergent sequences were highly enriched in these arrays, with 26 of the 304 clusters representing known cases; these are summarized in Table 5 ▸, along with information about space groups and sequence identities. When considering only non-enzymes (or non-proenzymes), 22 of the 124, or nearly one-fifth, are proteins found to form *in cellulo* arrays. The 26 clusters represent 17 well known biological categories, as discussed below. While the enrichment in *in cellulo* arrays in this set of lattice coincidences could arise if the crystal packing recapitulates the cellular association, not all associations have relevant evidence from *in cellulo* imaging and characterization.

#### 3.3.1. Ferritin and bacterioferritin

Under conditions of iron overload, ferritin can accumulate in cells and assemble into arrays in a strategy to safely sequester excess iron in a compact, inert form. The building block of the arrays is a 24-subunit ferritin nanocage capable of mineralizing thousands of Fe atoms. Ferritin paracrystalline arrays have been documented in hepatocytes, Kupffer cells and tissue macrophages in pathological states such as hereditary hemochromatosis and transfusion hemosiderosis, within lysosomes or specialized siderosomes (Massover, 1993 ▸). A closely related strategy is observed in bacteria through bacterioferritin, a homologous iron-storage protein that likewise assembles into a 24-subunit nanocage but incorporates haem groups at subunit interfaces.

#### 3.3.2. Woronin bodies

Woronin bodies are specialized, membrane-bound organelles found exclusively in filamentous fungi, such as *Neurospora crassa*, where they prevent cytoplasmic leakage following cell damage. Hex-1, the major protein component of Woronin bodies, self-assembles into crystalline cores within these organelles, providing the mechanical rigidity necessary for rapid pore sealing (Yuan *et al.*, 2003 ▸).

#### 3.3.3. Parasporal crystals

Bacterial δ-endotoxins (Cry toxins) are highly specific insecticidal pore-forming proteins that facilitate propagation of the bacterium by killing susceptible insect larvae after ingestion, thereby converting the host carcass into a nutrient-rich environment for spore germination and proliferation. The proteins are produced in extremely large quantities during sporulation and are packed into crystals to be stored at high local concentration without being toxic to the bacterium itself. The crystals only dissolve in the alkaline pH and protease-rich environment of an insect midgut. Electron microscopy and X-ray diffraction studies have extensively characterized these *in vivo* crystals, which can reach micrometre size (Jisha *et al.*, 2013 ▸).

#### 3.3.4. Charcot–Leyden crystals

Human galectin-10/Charcot–Leyden crystal protein is abundantly expressed in eosinophils. *In vivo*, it forms distinctive bipyramidal crystals: Charcot–Leyden crystals (CLCs). CLCs are a feature of asthma, are often found in parasitic diseases due to the influx of eosinophils and can be found in some haematological malignancies. These crystals are highly ordered and are easily identifiable by light and electron microscopy in tissue biopsies (Persson *et al.*, 2019 ▸).

#### 3.3.5. Pseudo-Charcot–Leyden crystals

Ym1/Ym2 crystals in mice produce pseudo-Charcot–Leyden crystals (Ym1/Ym2 crystals) from chitinase-like proteins. These proteins are not homologous with galectin-10. However, both types of crystals are associated with type 2 immunity and, like CLCs, can be found in the context of allergic reactions and parasitic infections (Aegerter *et al.*, 2021 ▸).

#### 3.3.6. Cypovirus polyhedra

Cypoviruses (cytoplasmic polyhedrosis viruses) are a genus of insect-infecting viruses belonging to the family *Reoviridae*. They possess a segmented double-stranded RNA genome and have non-enveloped, icosahedral capsids. Cypoviruses replicate exclusively in the cytoplasm of midgut epithelial cells, primarily in insect larvae from the order Lepidoptera. A distinctive feature of cypoviruses is their formation of occlusion bodies, in which crystalline polyhedrin proteins embed the viral particles, stabilizing and protecting them to enhance environmental persistence and transmission (Chen *et al.*, 2011 ▸).

#### 3.3.7. Baculovirus polyhedra

Baculoviruses are large, double-stranded DNA viruses within the family Baculoviridae, also known for infecting insect larvae from the order Lepidoptera. These viruses have a circular dsDNA genome and a distinctive rod-shaped enveloped nucleocapsid. Unlike cypoviruses, baculoviruses replicate in the nucleus of infected cells. They also produce occlusion bodies that encase virions (Chiu *et al.*, 2012 ▸).

#### 3.3.8. Entomopoxvirus spindles

Fusolin is a protein that forms part of ‘spindles’, crystalline structures produced by entomopoxviruses (EPVs). These spindles enhance viral infection by disrupting the insects’ peritrophic membrane (PM), a protective barrier in the gut. This disruption allows the virus to pass through the PM and reach the midgut epithelium, the primary site of infection (Mitsuhashi *et al.*, 2007 ▸).

#### 3.3.9. Prorenin

Prorenin is the inactive precursor of renin, an enzyme in the renin–angiotensin–aldosterone system (RAAS), which regulates blood pressure, electrolyte balance and fluid homeostasis. Synthesized primarily in the juxtaglomerular cells of the kidney, prorenin can stored intracellularly in dense granules and released into the circulation under conditions of physiological demand (Galen *et al.*, 1984 ▸). Prorenin crystals, which may have the same form as the deposited structures (Yan, 2017 ▸), have been observed in the cytoplasm of human tissue biopsies (Squires *et al.*, 1984 ▸), including from a patient with a juxtaglomerular cell tumour (Kuten *et al.*, 2004 ▸).

#### 3.3.10. Annexins

Annexin V is a phospholipid-binding protein consisting of a single annexin core domain, with a role in membrane-related processes such as membrane repair, exocytosis, endocytosis and apoptosis. It is a monomer in solution, but when bound on the cytoplasmic face of phospholipid membranes forms trimers that assemble into highly ordered 2D arrays in a calcium-dependent manner (Olofsson *et al.*, 1994 ▸).

#### 3.3.11. Bacterial microcompartment shell proteins

Bacterial microcompartments (BMCs) are organelles found in many bacteria that spatially organize specific metabolic pathways without using lipid membranes. BMC-H (hexameric) shell proteins are structural components of BMCs, forming flat, hexameric tiles that assemble into the proteinaceous shells of these organelles. EutM and CsoS1A are canonical BMC-H proteins that share a conserved fold consisting of a single BMC domain and form symmetric hexamers with small central pores that regulate metabolite diffusion. Despite their structural similarity, they occur in distinct functional contexts: EutM is a component of the ethanolamine-utilization (Eut) metabolosome, which catabolizes ethanolamine in enteric bacteria, whereas CsoS1A is found in α-carboxysomes, specialized BMCs used by autotrophic bacteria for CO_2_ fixation via the Calvin cycle (Trettel *et al.*, 2024 ▸).

#### 3.3.12. Microbial rhodopsin

Bacteriorhodopsin is a light-driven proton pump embedded in the purple membrane of halophilic archaea, where it converts photon energy directly into a transmembrane proton gradient. It forms exceptionally well ordered 2D crystalline arrays in native membranes, which made it a model system for electron crystallography and early atomic-resolution studies of membrane-protein structure. Cruxrhodopsin-3 from *Halo­arcula vallismortis* clusters with bacteriorhodopsin and can also oligomerize as trimers that laterally associate within the lipid bilayer to form extended, hexagonally ordered 2D arrays, analogous to the classic purple membrane. The structure in the cluster, in space group *P*321, is related to the structures in the cluster that includes the first structure of bacterio­rhodopsin, PDB entry 1ap9 in space group *P*6_3_, with √CC*_I_* of 0.3 and NCDist of 3.5 Å, which is outside the clustering criteria used for this study but nevertheless is significant.

#### 3.3.13. Vicilin

Vicilins are plant seed storage proteins (7S globulins) that accumulate to extremely high concentrations during seed development and frequently organize into paracrystalline arrays within protein-storage vacuoles. Paracrystallinity allows vicilins to be stored in a compact, osmotically inert form while remaining readily mobilizable during germination, when proteolysis rapidly dismantles the ordered assemblies. Paracrystalline organization represents a functional storage strategy: structural order is sufficient to stabilize and condense large protein reserves, yet deliberately imperfect to permit efficient biological turnover.

#### 3.3.14. RecA

RecA is a bacterial DNA-repair protein that plays a role in homologous recombination. *In vivo*, RecA polymerizes along single-stranded DNA to form a nucleoprotein filament in bacterial cells under stress. The RecA–DNA filament is considered a dynamic, functional crystal-like structure, exhibiting helical symmetry with a characteristic pitch (∼95 Å) and repeating subunits that align along the DNA backbone (Nishinaka *et al.*, 2007 ▸).

#### 3.3.15. Amyloidosis

Amyloid light-chain amyloidosis is a polymerization-driven disease where misfolding events in immunoglobulin light chains, from an abnormal clone of plasma cells in the bone marrow, lead to the formation of small amyloid nuclei, recruitment of further chains and rapid fibril elongation. This devastating process makes the disease progression self-amplifying. The structures of highly fibrillogenic light-chain variable-domain mutants shows molecules packing into extended arrays dominated by β-sheet-mediated contacts, producing linear, zipper-like interactions. These crystal-packing arrangements are widely interpreted as the fibrillar assemblies that accumulate in amyloidosis (Luna-Martínez *et al.*, 2017 ▸).

#### 3.3.16. Phospholipase A_2_

Phospholipase A_2_ (PLA_2_) is a small, secreted enzyme that hydrolyses phospholipids in a calcium-dependent manner, releasing fatty acids and lysophospholipids and acting as a potent toxin. Because this activity would be catastrophic inside the producing cell, PLA_2_ must be rendered inactive prior to secretion. PLA_2_ is stored in dense secretory granules, as observed ultrastructurally in venom glands. Reversible oligomerization and quasi-ordered packing provide an effective strategy to neutralize enzymatic activity by restricting access to membranes and substrates. Although true intra­cellular crystals of PLA_2_ have not been demonstrated, the combination of ready crystallizability, extreme local concentration in the cells and known venom-granule biology strongly supports storage in highly ordered granular assemblies that suppress activity until venom secretion.

## 4. Discussion

Multiple crystal forms sharing a lattice coincidence can appear across different investigations of the same or similar systems by different crystallographers separated by time and laboratory. Our PDB-wide analysis shows that lattice coincidences are common amongst deposited structures; the number of distinct lattice clusters is approximately half the total number of PDB entries, of which only half are identifiable through structure-overlap tests.

Our software *Scotty* can rapidly scan the PDB archive for closely and distantly related crystal forms. In general, the tolerances used for probing lattice coincidences for biological relevance can be broad, as although most matches suitable for DFFT phasing are made with NCDist < 3 Å, significant matches are made up to NCDist = 5 Å. Likewise, thresholds for √CC_*I*_ (default 0.4) can be lowered, for example to 0.3, to consider matches that may be of interest when other supporting information suggests that the relationships are biologically relevant. However, care should be taken with short sections of DNA and coiled coils, where convergences in cell dimensions and strong helical diffraction patterns can give high √CC_*I*_ regardless of sequence.

In the future, we aim to make the *Scotty* search available through server access. This will synchronize the search with PDB updates and further improve access.

Biological interpretation of lattice coincidences for any given use case must be approached with a nuanced understanding of the biology of the system under study. For example, from our own work, the identification of prorenin amongst the number of structures in the isomorphous set of distant homologues may indeed have biological relevance. Prorenin is the inactive precursor of renin, an enzyme in the renin–angiotensin–aldosterone system (RAAS), which regulates blood pressure, electrolyte balance and fluid homeostasis. Synthesized primarily in the juxtaglomerular cells of the kidney, prorenin can be stored intracellularly in dense granules, activated enzymatically to mature renin and released into the circulation under conditions of physiological demand (Galen *et al.*, 1984 ▸). Prorenin crystals have been observed in the cytoplasm of human tissue biopsies (Squires *et al.*, 1984 ▸). The identification of prorenin in our survey, which has a high proportion of biologically relevant paracrystalline assemblies, supports the proposition that the characterized prorenin crystals are likely to be those seen *in cellulo*. We aim to pursue experimental confirmation of this hypothesis with the aid of recent advances in cryo-electron tomography, *in situ* diffraction techniques and correlative light and electron microscopy (CLEM), which are now starting to enable characterization of these protein crystals within challenging native cellular environments (Yang *et al.*, 2024 ▸). However, this work remains technically difficult, not least in the difficulty of obtaining and preparing suitable tissue for analysis.

Surprisingly, our limited survey of the PDB archive, restricted to the subset of those entries with only one protein entity in the asymmetric unit, showed that lattices conserved through evolution were highly enriched in structures known to naturally form 3D, 2D and 1D helical assemblies. Note that our survey restrictions excluded known paracrystalline arrays such as insulin, which after processing of proinsulin is annotated as chain *A* and chain *B*, and hence does not meet the criteria for inclusion in the survey.

Our survey focused on clusters of proteins with divergent sequences as a proxy for biological significance in the absence of specific domain knowledge. Conservation of crystal packing between different crystal forms of the same or closely related proteins is also of interest. Given the speed and ease of crystallographic structure solution at modern synchrotron facilities, we would encourage crystallographers to consider solving all crystal forms observed in crystallization trials in order to extract any biologically relevant information that may be present in lattice coincidences.

We hope that those with specialized knowledge of interesting systems will find promising research questions, within and without the scope of this survey, with this new research tool.

## Supplementary Material

Supplementary Table S1. DOI: 10.1107/S2059798326005723/nz5022sup1.pdf

## Figures and Tables

**Table 1:** Distribution of space groups per lattice cluster Macromolecules (more than 34 residues) are capped at a maximum of four space groups per cluster. The highly inflated clusters (containing five to eight space groups) consist entirely of short DNA and α-helical peptides in which end-to-end packing artefacts distort the structural analysis.

Space groups per cluster	Macromolecules	DNA/peptide helices	Total clusters
2	1351	64	1415
3	83	17	100
4	8	7	15
5	0	3	3
6	0	1	1
7	0	3	3
8	0	1	1
Total	1442	96	1538

**Table 2:** Historical space-group propensities in the PDB for three most common space groups

Study/citation	Table	Count	*P*2_1_2_1_2_1_ (%)	*P*2_1_ (%)	*C*2 (%)
Padmaja *et al.* (1990 ▸)	6	208 unique crystal structures	26.9	10.6	12.9
Wukovitz & Yeates (1995 ▸)	1	245 unique monomeric proteins	36.1	11.9	6.1
Andersson & Hovmöller (2000 ▸)	1	7384 curated sequences with >15 residues	22.9	13.6	8.9
Chruszcz *et al.* (2008 ▸)	3	9081 nonredundant sequence subset	21.1	15.6	9.4
Gaur (2021 ▸)	1	20655 curated nonredundant proteins; 11 categories	15.3–26.0	10.2–24.1	9.3–10.3
This study	S1	88242 lattice clusters with >34 residues	20.7	17.8	10.8

**Table 3:** Space-group propensities binned by presence of point-group symmetry operations for lattice clusters of single protein entity structures selected from the PDB 20261010 snapshot as described in the text Space groups without point-group symmetry operations (Schönflies *C*_2_, *C*_3_, *C*_4_, *C*_6_, *D*_2_, *D*_3_, *D*_4_, *D*_6_, *T* or *O*) are very significantly underrepresented. Note that *P*2_1_2_1_2_1_ and *P*2_1_, the two most common space groups, are included in the list of underrepresented space groups. Amalgamated data from Table 4 ▸ for the PDB 20260101 snapshot are included for comparison.

	Clusters	Screw†	% Screw	Non-screw	% non-screw
Monomeric	3628	1454	40.1	2174	59.9
Oligomeric	2631	227	8.6	2404	91.4
20260101	882420	39644	45.0	48500	55.0

† Space groups with only screw rotations or no proper rotations: *P*1, *P*2_1_, *P*2_1_2_1_2_1_, *P*4_1_, *P*4_2_, *P*4_3_, *P*3_1_, *P*3_2_, *P*6_1_, *P*6_5_, *P*6_2_, *P*6_4_, *P*6_3_

**Table 4:** Paracrystallinity in the PDB Clusters from the PDB survey of structures containing only one protein entity and one molecule in the asymmetric unit (in at least one of the members of the cluster), with sequence-divergent members and with known biologically observed crystalline or paracrystalline arrays. Sequence identities for the representatives in the cluster are shown as superscripts except where the sequences are identical to the reference. At least one sequence in the cluster is below 95% sequence identity to the reference.

	Name of paracrystalline array	No. in cluster	Space groups in cluster	Reference for sequence identities	Representative structures in cluster
1	Amyloidosis	2	*I*2_1_2_1_2_1_, *I*4_1_22	1tpe	5ir3, 5jpj^(87%)^
2	Annexin	7	*H*3	1tpe	1ala^(78%)^, 1avr, 1hvd^(99%)^, 1hve^(99%)^, 1hvf^(99%)^, 1sav, 1yii^(78%)^
3	Annexin	19	*P*1, *H*3	1tpe	1anx^(92%)^, 1hvg^(91%)^, 2ran, 2xo2^(90%)^, 2xo3^(92%)^
4	BMC-H shell	2	*P*6	1tpe	3mpy, 7mgp^(51%)^
5	BMC-H shell	4	*P*6	1tpe	2ewh^(53%)^, 2g13^(53%)^, 3dnc^(54%)^, 5d6v
6	BMC-H shell	2	*P*6_3_	1tpe	3n79, 3pac^(92%)^
7	BMC-H shell	5	*P*6_3_, *P*6, *P*2_1_	1tpe	3h8y, 4liw^(47%)^, 7mn4^(45%)^, 7mpx^(47%)^, 8yxu^(97%)^
8	Baculovirus polyhedra	9	*I*23	1tpe	2wux^(99%)^, 2wuy, 3jvb^(81%)^, 3jw6^(99%)^, 5g0z^(55%)^, 5g3x^(55%)^, 5mnd^(56%)^, 6s2o^(55%)^, 6yng^(55%)^
9	Charcot–Leyden crystals	38	*P*6_5_22	1tpe	7xxu^(79%)^, 7xxx^(80%)^
10	Cry toxin	8	*C*222_1_	1tpe	1dlc, 1ji6^(67%)^, 4qx0, 4qx1, 4qx2, 4qx3, 6lfp, 7ear^(98%)^
11	Cry toxin	2	*P*4_1_2_1_2	1tpe	4w8j, 8w7n^(84%)^
12	Cypovirus polyhedra	4	*I*23	1tpe	5a8s, 5a8t, 5a9a^(36%)^, 5a9c
13	Cypovirus polyhedra	35	*I*23	1tpe	2oh5, 5a9p^(86%)^, 5gqj, 6lee, 7wyr, 8j2q^(95%)^, 8wlg^(96%)^, 8x8v^(95%)^
14	Ferritin	17	*F*432	1tpe	7vp8^(46%)^, 8wpt
15	Ferritin	224	*F*432, *F*23	1tpe	1gwg, 3es3^(52%)^, 3ka3^(58%)^, 3ka6^(58%)^, 3ka8^(58%)^, 3ka9^(58%)^, 3sh6^(57%)^, 3shx^(57%)^, 4dyx^(53%)^, 4dz0^(52%)^, 4lpm^(57%)^, 4lpn^(57%)^, 4mjy^(58%)^, 5lg2, 5wpn^(54%)^, 5xhm^(57%)^, 5xho^(58%)^, 5z8u^(52%)^, 6jef^(98%)^, 6ke2^(54%)^, 6trz, 6wyf^(54%)^, 7bd7, 7vio^(98%)^, 8b7o^(54%)^
16	Ferritin	13	*F*432, *F*23	1tpe	3r2h^(41%)^, 4am2^(62%)^
17	Ferritin	30	*I*23, *I*432, *I*222, *H*32	1tpe	2fg4, 3kxu^(95%)^, 3vnx^(36%)^, 5cmr^(56%)^, 5lg8, 5up8^(56%)^, 6j4m^(42%)^, 6kzy^(48%)^, 6ljg^(53%)^, 6lpd^(48%)^, 7dlb^(53%)^, 7xz4^(43%)^, 8w91^(52%)^
18	Microbial rhodopsin	5	*P*321	1tpe	7zou, 7zov, 7zow, 7zoy, 8h79^(34%)^
19	PLA_2_	6	*C*2, *H*3, *R*32	1tpe	1dpy, 1fe5^(91%)^, 1g0z^(81%)^, 1g2x^(84%)^, 1u4j^(81%)^, 2osn^(84%)^
20	PLA_2_	19	*P*4_1_	1tpe	1ln8^(98%)^, 1mh7^(84%)^, 1t37^(97%)^, 1yxh^(87%)^, 2osh^(93%)^, 3gci^(97%)^
21	PLA_2_	57	*P*4_3_	1tpe	1q6v^(96%)^, 2do2^(97%)^, 2pvt^(91%)^
22	Prorenin	3	*I*4_1_	1tpe	4amt^(70%)^, 5mkt, 5mlg^(85%)^
23	Spindles	4	*P*4_1_2_1_2	1tpe	4ow5, 4x27, 4x29, 4yn1^(59%)^
24	Vicilin	2	*H*32	1tpe	5cad, 5vf5^(78%)^
25	Woronin bodies	14	*P*6_5_22	1tpe	7asi, 8qlu^(69%)^, 8qlw^(78%)^, 8qlx^(74%)^, 8qnu^(79%)^
26	Pseudo-Charcot–Leyden crystals	6	*P*2_1_	1tpe	1e9l, 1vf8, 8p8q, 8p8r, 8p8s^(90%)^, 8p8t^(90%)^

**Table 5:** Comparison of contact interfaces for two keto–acid reductoisomerases with a lattice coincidence. The keto–acid reductoisomerases from *M. thermolithotrophicus* (PDB entry 7q03) and *C. jejuni* (PDB entry 7lat) are annotated as a dodecamer and dimer, respectively Both have one protein molecule in the asymmetric unit in space group *I*23, with the lattices coincident after a re-indexing (−*h*, *l*, *k*). Two symmetry relationships are used to build interfaces for the dodecameric assembly. For each interface, data from the *PISA* and *EPPIC* analyses are shown side by side. The dominant interface (row 1) is strong for both lattices and represents an interlocked dimer. The secondary interface (row 2) is regarded as significant by *PISA* only for the thermophile, although the differences are only marginal.

	*M. thermolithothrophicus* (PDB entry 7q03, dodecamer)					*C. jejuni* (PDB entry 7lat†, dimer)				
	*PISA* ‡				*EPPIC* §	*PISA* ‡				*EPPIC* §
Symmetry	Area	Δ*G*	*N*_HB_	*N*_SB_	Core-surface	Area	Δ*G*	*N*_HB_	*N*_SB_	Core-surface
*x*, −*y*, −*z*	6123	−94.7	38	46	BIO [−6.04]★	4667	−90.7	20	10	BIO [−6.24]★
*z*, *x*, *y*	501	−4.2	4	2	XTAL [0.49]★	459	−1.5	2	1	XTAL [−0.25]★

† Results are shown for PDB entry 7lat transformed to the indexing of PDB entry 7q03.

‡ *PISA*: area is give in Å^2^ and Δ*G* in kcal mol^−1^; *N*_HB_ is the number of hydrogen bonds and *N*_SB_ is the number of salt bridges.

§ *EPPIC*: the prediction of assembly interface (BIO) versus crystal contact (XTAL) is qualified with a *Z*-score of the sequence entropy of core residues at 70% burial versus random samples of all surface residues [in brackets], with a gold star for high confidence and grey star for medium confidence, as per the *EPPIC* markup.

## Data Availability

*Scotty* will be made available through the *Phenix* and *CCP*4 software packages.
