# Supplementary material for: Scotty: lattice coincidences in the Protein Data Bank
Source: Acta Crystallogr D Struct Biol. 2026 Jun 24;82(Pt 7):824–35. doi: 10.1107/S2059798326005723 (PMC13317678; doi:10.1107/S2059798326005723)
Supplement: Supplementary file 1 [file d-82-00824-sup1.pdf]

STRUCTURAL BIOLOGY

Volume 82 (2026)

Supporting information for article:

Scotty: lattice coincidences in the Protein Data Bank

Airlie J. McCoy, Lawrence C. Andrews, Herbert J. Bernstein and Randy J. Read

Table S1 Crystallographic space group propensities in the PDB for 88242 lattice clusters with primary sequence over 15 residues.

rank	space group	count	percentage
1	$P2_12_12_1$	18257	20.69
2	$P2_1$	15707	17.80
3	C2	9483	10.75
4	$P22_12_1$	5168	5.86
5	P1	4564	5.17
6	$C222_1$	4474	5.07
7	$P4_12_12$	2845	3.22
8	$P4_32_12$	2774	3.14
9	$P3_121$	2523	2.86
10	$P3_221$	2512	2.85
11	I222	1424	1.61
12	$P6_122$	1258	1.43
13	$P6_522$	1214	1.38
14	R32	1067	1.21
15	R3	1018	1.15
16	$P6_5$	927	1.05
17	$P6_1$	870	0.99
18	$P3_1$	635	0.72
19	$P6_3$	626	0.71
20	$P3_2$	617	0.70
21	$P4_3$	574	0.65
22	$P4_1$	568	0.64
23	$I4_122$	559	0.63
24	$P6_322$	558	0.63
25	$P4_22_12$	525	0.60
26	I422	516	0.58
27	I4	457	0.52
28	P321	366	0.41
29	$P6_222$	347	0.39
30	$P2_13$	346	0.39
31	$P6_422$	339	0.38
32	$P42_12$	330	0.37
33	I23	325	0.37
34	$I4_1$	308	0.35
35	$I2_12_12_1$	302	0.34
36	P2	302	0.34
37	$P4_322$	287	0.33
38	$P4_122$	278	0.32
39	C222	251	0.28
40	$P6_2$	233	0.26
41	$P6_4$	232	0.26
42	$I2_13$	193	0.22
43	F222	173	0.20
44	$P222_1$	167	0.19
45	P4132	147	0.17
46	P3	131	0.15
47	$P3_112$	123	0.14
48	P6	122	0.14
49	$P3_212$	116	0.13
50	$P4_332$	110	0.12
51	P4	108	0.12
52	P622	102	0.12
53	$I4_132$	87	0.10
54	I432	84	0.10
55	F432	78	0.09
56	$P4_222$	77	0.09
57	$P4_2$	74	0.08
58	P23	65	0.07
59	F23	60	0.07
60	$F4_132$	59	0.07
61	P432	44	0.05
62	P422	42	0.05
63	$P4_232$	26	0.03
64	P312	26	0.03
65	P222	10	0.01
